# Association of novel MUC16, MAP3K15 and ABCA1 mutation with giant congenital melanocytic nevus

**DOI:** 10.1186/s41065-022-00247-8

**Published:** 2022-09-09

**Authors:** Renpeng Zhou, Qirui Wang, Jialin Hou, Danru Wang, Yimin Liang

**Affiliations:** Department of Plastic and Reconstructive Surgery, Shanghai Ninth People’s Hospital, Shanghai JiaoTong University School of Medicine, 639 Zhi Zao Ju Road, Shanghai, 200011 People’s Republic of China

**Keywords:** Giant congenital melanocytic nevus (GCMN), MUC16, MAP3K15, ABCA1, Whole-exome sequencing (WES)

## Abstract

**Background:**

Giant congenital melanocytic nevus (GCMN) is the benign nevomelanocytic proliferation. Mutations in NRAS have been previously detected in GCMN, but mutations in BRAF are generally lacking in the Chinese population. Mutated genes in this disease can estimate the risk of malignant transformation in GCMN. Therefore, it is worth investigating the genetic information of GCMN.

**Methods:**

Here, we presented two cases of GCMN of the upper extremities. The clinical and histological data were analyzed. The whole exome sequencing (WES) was performed to investigate the mutational profile of peripheral venous blood (PB), normal skin (NS), small melanocytic nevus (SMN), deep penetrating and non-penetrating GCMN (dPGCMN and nPGCMN).

**Results:**

We showed a reduction in the circumference of involved upper extremities in both patients. The clinical and histopathological data indicated the reduction of adipose tissue associated with the invasion of GCMN. The WES data revealed that MUC16, MAP3K15 and ABCA1 were novel potential candidate genes for the disease as well as biomarkers for predicting malignant transformation.

**Conclusion:**

The MUC16, MAP3K15 and ABCA1 may serve as novel biomarkers for predicting malignant transformation and targets for the diagnoses and therapy for the GCMN.

## Introduction

Giant congenital melanocytic nevus (GCMN) is usually defined as a melanocytic lesion presented at birth, whose diameters will reach ≥20 cm in diameter in adulthood [1, 2]. The incidence of GCMN is estimated at < 1:20,000 neonates [1]. GCMN usually presents as well-defined brown lesions with a flat or papillary surface and hirsutism. It is now well accepted that GCMN is closely associated with malignant melanoma [3], but the precise extent of the risk remains unknown. The lifetime risk of melanoma in patients with GCMN has been reported to be between 10 and 15% [4, 5]. In addition to the high rate of malignancy, the severe aesthetic defects of GCMN impose a heavy psychological burden on patients, especially in the young age groups [1].

Congenital melanocytic nevus is primarily a clinical diagnosis. However, it is not effective to determine whether GCMN is at potential risk of malignant transformation. Therefore, clarification of GCMN genetic alterations can improve the accuracy of clinical diagnosis and assessment of the risk of malignant transformation. A current study suggests that the formation of GCMN is directly associated with NRAS gene mutations, which leads to sustained activation of the mitogen-activated protein kinase (MAPK) pathway and promotion of melanophore proliferation [6]. Nevertheless, there is no evidence that the high rate of malignant transformation in GCMN is closely associated with NRAS mutations [7]. Furthermore, mutations in BRAF^V600E^, a major oncogene associated with melanoma, may increase the risk of malignancy in GCMN [8], but such mutations are absent in the Chinese population [9]. Hence, it is necessary to search for mutated genes associated with the transition from GCMN to malignant melanoma in the Chinese population.

Here, we reported two patients with upper extremity GCMN. We performed whole exome sequencing (WES) to investigate the mutated genes of interest in both patients. We identified MUC16, ATP-binding cassette transporter A1 (ABCA1) and mitogen-activated protein kinase kinase kinase 15 (MAP3K15) as potential critical mutated genes closely associated with GCMN. To the best of our knowledge, this is the first report of an upper extremity GCMN genetic study in Asian.

## Method

Two children with upper extremity GCMN were included in this study. The study was approved by the Ethics Committee of Shanghai Ninth People’s Hospital, Shanghai Jiao Tong University School of Medicine. Prior to the study, we obtained written informed consent from each patient’s guardians regarding participation in this research. Based on the expert consensus and standardized classification of skin features in congenital melanocytic nevi, we found that two patients met the GCMN criteria [10]. Final clinical data were recorded, including detailed medical history, physical examination data, histopathological and genetic analysis results.

**Fig. 1:**
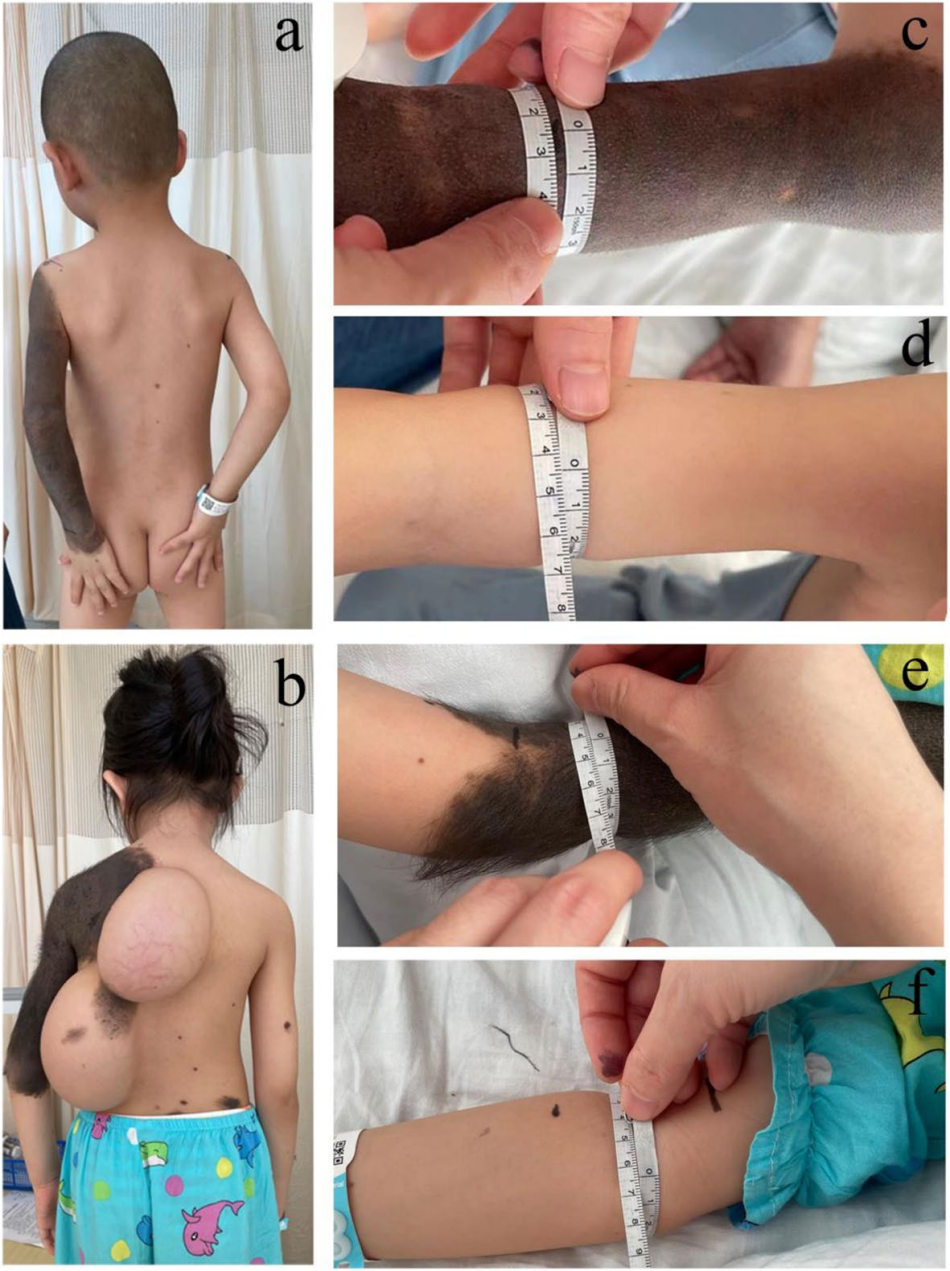
**a** Clinical presentation of patient 1#: The GCMN lesion was located on the left upper extremity, with clear borders, no hirsutism, and brownish-black color. **b** Clinical presentation of patient 2#: The GCMN lesion was located above the elbow and left shoulder of the left upper extremity, with a well-defined, dark, hairy lesion. **c** The upper extremity of patient 1# with GCMN was measured for circumference using a soft ruler. **d** The healthy upper extremity of patient 1# was measured for circumference using a soft ruler. **e** The upper extremity of patient 2# with GCMN was measured for circumference using a soft ruler. **f** The healthy upper extremity of patient 2# was measured for circumference using a soft ruler. GCMN: Giant congenital melanocytic nevus

Patient 1# was a 4-year-old boy who presented with GCMN in the left upper extremity (Fig. 1a). The lesions of GCMN had well-defined borders, no hirsutism, and a brownish-black color bias. This patient did not present with scattered melanocytic nevi. Patient 2# was a 5-year-old girl who presented with GCMN lesions on the left upper extremity above the elbow and the left shoulder. The GCMN lesions were well-defined, dark and hirsute (Fig. 1b). Scattered small and medium-sized melanocytic nevi were found on the back and right upper extremity of the patient.

Both patients underwent surgical excision of the lesion and flap grafting to cover the defects. Intraoperatively, we found that the GCMN of patient 2# extended into the muscular layer. Since the characteristics of this patient’s deep penetrating GCMN were very similar to deep penetrating nevi (DPN) [11], we divided her GCMN tissue into two parts: deep penetrating (dPGCMN) and non-penetrating (nPGCMN). In order to investigate the histological and genetic changes of GCMN in both patients, samples of excised GCMN tissue and adjacent normal skin tissue were taken for subsequent analysis. Additionally, we collected small melanocytic nevus (SMN) tissue from patient 2#. In addition to the tissue samples, peripheral venous blood (PB) samples were collected from the patients. The GCMN and normal skin tissues were fixed in 10% formaldehyde solution and then embedded in paraffin. The embedded tissues were sectioned and stained with hematoxylin-eosin using routine procedures. Pathological analysis was performed by two experienced pathologists. Other tissue specimens were preserved in liquid nitrogen and used for DNA extraction.

In brief, DNA from all tissues and blood samples described above was extracted according to standard procedures. For each sample, 100–200 ng of genomic DNA was randomly fragmented to < 300 bp by sonication. The interrupted DNA fragments were end-repaired using End Prep Enzyme Mix. Next, Poly-A tails were added to the 3′ ends by A Tailing Mix and then purified. The results were amplified using Pre-Capture PCR primers. PCR products were validated with Qsep100 (Bioptic, Taiwan, China) and quantified with Qubit3.0. Next, a high-throughput sequencing library was constructed after exon capture and PCR enrichment steps. WES was performed using Illumina HiSeq. Finally, the raw exome sequencing data were analyzed by bioinformatics analysts of GENEWIZ Inc.

We analyzed the results of WES. We divided the samples into six comparison groups: patient 1# normal skin tissue versus peripheral venous blood (NS vs PB) and GCMN versus peripheral venous blood (GCMN vs PB); patient 2# normal skin tissue versus peripheral venous blood (NS vs PB), deep penetrating GCMN versus peripheral venous blood (dPGCMN vs PB), non-penetrating GCMN versus peripheral venous blood (nPGCMN vs PB), and small melanocytic nevi versus peripheral venous blood (SMN vs PB). We also identified mutated genes associated with deep penetration by comparing the differences between dPGCMN and nPGCMN in patient 2#. We used an online website to make pathogenicity predictions for the mutated genes screened. Three different algorithms, SIFT (http://sift.jcvi.org/), Polyphen2 (http://genetics.bwh.harvard.edu/pph2/) and MutationTaster (http://www.mutationtaster.org/), were used to predict the effect of mutated genes on protein function and potential pathogenicity.

## Results

**Fig. 2:**
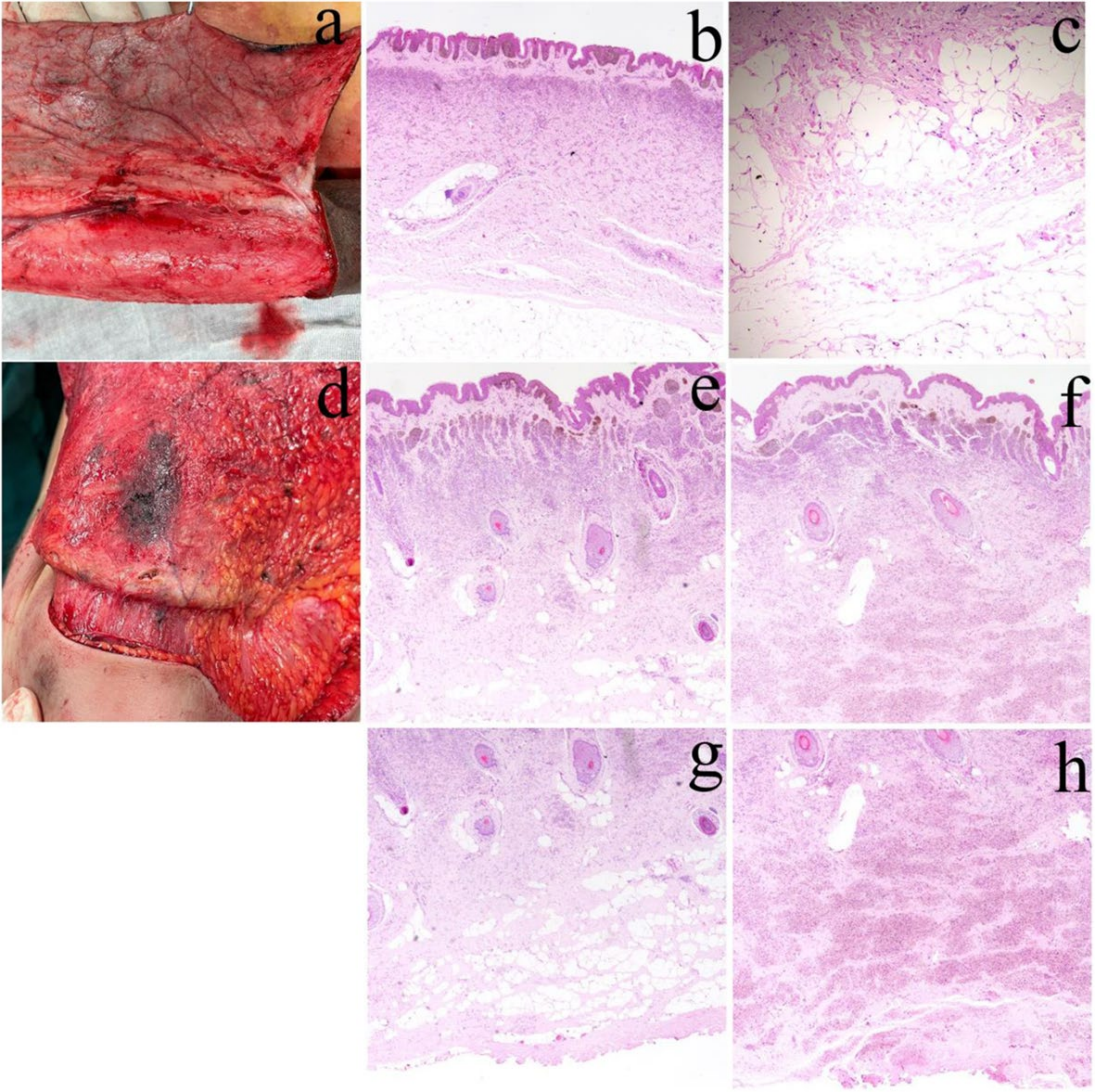
**a** Gross appearence of a GCMN lesion in patient 1# during excision. **b** Hematoxylin-eosin staining of GCMN tissue from patient 1# (original magnification × 100). **c** Hematoxylin-eosin staining of adipose tissue in the deeper layers of the GCMN in patient 1# (original magnification × 400). **d** Gross appearence of a GCMN lesion in patient 2# during excision. **e** Hematoxylin-eosin staining of nPGCMN tissue of patient 2# (original magnification × 100). **f** Hematoxylin-eosin staining of dPGCMN tissue of patient 2# (original magnification × 100). **g** Hematoxylin-eosin staining of deep adipose tissue of patient 2# in the nPGCMN (original magnification × 100). **h** Hematoxylin-eosin staining of the deep tissue of patient 2# in the dPGCMN (original magnification × 100). The adipose tissue disappears and is replaced by melanocytes. GCMN: Giant congenital melanocytic nevus; nPGCMN: Non-penetrating giant congenital melanocytic nevus; dPGCMN: Deep penetrating giant congenital melanocytic nevus

The physical features of the GCMN lesions in patients 1# and 2# were as described above. Besides, we found that the circumference of the forearm with GCMN was smaller than the healthy side by about 2 cm in both patients (Fig. 1c and d). Based on the histological findings, the GCMN of patient 1# was a compound nevus with melanocytes aggregated at the epidermal and dermal junction (Fig. 2b and c). While the GCMN of patient 2# was a subcutaneous nevus with melanocytes aggregated in the dermis. However, melanocytes of GCMN in patient 2# penetratingly invaded into the muscle layer compared to patient 1# (Fig. 2d). At the histological level, normal adipose tissue was present in the deeper layers of GCMN in patient 1# and the nPGCMN in patient 2# (Fig. 2c and g). Interestingly, the adipose tissue was absent from the dPGCMN site in patient 2#, and the adipose tissue was replaced by aggregated melanocytes (Fig. 2h).

We performed WES analysis of DNA from skin lesion tissue, normal skin tissue and peripheral venous blood. After comparison with peripheral venous blood, both patient 1# and patient 2# showed mutations in NRAS and MUC16 genes in the GCMN. Furthermore, strikingly, dPGCMN tissues in patient 2# specifically displayed mutations in ABCA1 and MAP3K15 genes compared with other tissues, including the SMN and nPGCMN (Table 1). The occurrence of gene mutations in NRAS in GCMN has been widely noticed and recognized, so we explored for other three genes. Table 2 shows the mutation information of ABCA1, MAP3K15 and MUC16 in detail, including the nucleotide and amino acid change, exon positions. After validation by three pathogenicity prediction algorithms (MutationTaster, PolyPhen2 and SITF), these mutated genes may have crucial roles in the growth, invasion and malignant transformation of GCMN.

**Table 1:** Genetic mutations in different tissues of patient 1# and 2# compared to peripheral venous blood

Gene	Patient 1#		Patient 2#			
	NS vs PB	GCMN vs PB	NS vs PB	dPGCMN vs PB	nPGCMN vs PB	SMN vs PB
NRAS	0	1	0	1	1	0
ABCA1	0	0	0	1	0	0
MAP3K15	0	0	0	1	0	0
MUC16	0	1	0	1	1	0

*NS* vs *PB* normal skin tissue versus peripheral venous blood, *GCMN* vs *PB* GCMN versus peripheral venous blood, *dPGCMN* vs *PB* deep penetrating GCMN versus peripheral venous blood, *nPGCMN* vs *PB* non-penetrating GCMN versus peripheral venous blood, *SMN* vs *PB* small melanocytic nevi versus peripheral venous blood, *0* No mutations detected, *1* Mutations detected

**Table 2:** Details of mutant genes

Gene	Nucleotide change	Amino acid change	Exon	MutationTaster	PolyPhen2	SITF
ABCA1	c.6579C > G	p.I2193M	Exon49	disease causing	–	–
MAP3K15	c.3272 T > G	p.V1091G	Exon23	disease causing	–	Damaging
MUC16	c.40923C > T	p.F13641F	Exon59	disease causing	–	–

## Discussion

In the present study, we report two pediatric patients with upper extremity GCMN. Patient 1# had a GCMN that was a complex nevus with melanocytes aggregated at the epidermal-dermal junction. Patient 2# had a subcutaneous nevus that reached deep into the muscular layer, suggesting a higher GCMN invasive capacity in this patient. We evaluated the differences of GCMN tissue between the two patients at the histological and genetic levels.

At the histological level, it is speculated that the histological features of patient 2# may be a consequence of the continuous development of patient 1#. In the early stages (as in patient 1#), the melanocytes of GCMN remain mainly at the epidermal and dermal junction. In contrast, as the child grows (as in patient 2#), GCMN gradually infiltrates into the dermis and subcutaneous tissues (Fig. 2). As shown in Fig. 1, both patients exhibited a reduced circumference of the upper extremity with GCMN compared with the unaffected side. This phenomenon has been noticed in patients with GCMN of the extremities [12]. It has been hypothesized that this reduction in circumference may be due to the replacement of adipose tissue by nevus cells [12], but our results show that the adipose tissue in the deep layer of GCMN of patient 1# and the adipose tissue of nPGCMN of patient 2# were not replaced and the circumference was still reduced. Combining our histological results with previous studies [13], we suggest that the reduced circumference of the upper extremity with GCMN may be due to melanocyte infiltration of the dermis and destruction of collagen.

In addition, we found that a portion of GCMN tissue in patient 2# penetrated deep into the subcutaneous tissue to the muscular layer, a histological feature similar to DPN. DPN is a lesion that exhibits nested clusters of melanocytes infiltrating deeper into the dermis, which may exhibit the features of malignant melanoma [14]. Interestingly, combination of congenital and deep penetrating nevus has been reported in the previous studies [15], whereas GCMN combined with DPN has not been reported. We believe that the histopathological features of patient 2# presenting with DPN are the result of further development of GCMN. Therefore, this histopathological feature suggestes that GCMN in patient 2# may appear to mimic the biological behavior of melanoma, such as invasion of surrounding tissues. However, it is difficult to determine whether there is an increased risk of cancer in GCMN from histopathological features alone, which requires a combination of molecular diagnostics for accurate prediction.

To characterize the genetic alterations of GCMN, we performed a genetic analysis of GCMN tissues, small melanocytic nevus tissues, normal skin tissues and peripheral venous blood of patients utilizing WES. By comparing with exon sequences of peripheral blood, we found that GCMN tissues showed different mutational characteristics in terms of individual, size and depth of infiltration. By screening, we identified four significantly mutated genes, and NRAS, MUC16, ABCA1 and MAP3K15. In our results, the NRAS gene mutation was consistent with those reported previously [6, 16]. This result validates the important role of this gene in the development of GCMN, and we will not discuss it in depth here. Interestingly, we did not find mutations in BRAF in the GCMN of these patients, and it has been suggested that mutations in BRAF are generally lacking in GCMN patients in the Chinese population [9]. Clearly, at least in the Chinese population, BRAF mutations, which are frequent in melanoma and GCMN with a high risk of malignant transformation, cannot be used as a valid marker to predict malignant transformation. In addition, neither NS nor SMN had mutations in these four genes compared to PB, which indicated that SMN does not have the same risk of malignancy as GCMN.

Based on our WES results, we accidentally identified a mutation in MUC16. MUC16 is a member of the mucin family, also known as tumor-associated antigen CA125, and is overexpressed in more than 80% of ovarian cancers [17]. Importantly, in melanoma, MUC16 is one of the critical mutated genes and is expected to be an alternative biomarker of tumor mutation burden [18]. In this study, MUC16 was mutated in both cases. Compared with BRAF, we suggest that MUC16 may be a more suitable biomarker for detecting the transformation of GCMN to melanoma in the Chinese population. However, given the limited number of cases, the potential value of the gene needs to be confirmed with more samples. ABCA1 is a cell membrane protein that regulates cholesterol efflux and triggers many signaling pathways by interacting with apolipoprotein receptors [19]. Moreover, ABCA1 is abundantly expressed in adipose tissue and contributes to HDL biogenesis [20]. Recently, Cuffe et al. confirmed that ABCA1 is critical for adipocyte biogenesis and lipid accumulation and deletion of ABCA1 leads to smaller adipocytes and lower fat pad weight and lower body weight in mice [21]. Therefore, mutations of ABCA1 in dPGCMN of patient 2# may contribute to the reduction of subcutaneous fat. MAP3K15, also known as ASK3, is a member of the MAPK family. Several studies have found that members of the MAPK family function in a protein kinase signaling cascade and that MAP3K proteins play a role in regulating apoptosis [22]. It was found that MAP3K15 may be involved in tumorigenesis and development [23]. Importantly, our study identified mutations of the MAP3K15 gene in the dPGCMN tissue of patient 2#, which may have impaired the normal apoptotic program of melanocytes leading to uncontrolled cell proliferation. Moreover, the current view speculates that an abnormal state of sustained activation of the MAPK pathway promotes the continued proliferation of melanocytes, which lays the foundation for the continuous infiltration of GCMN into the surrounding tissues. In addition, by comparing the mutation results between dGCMN and nGCMN, we found that only dGCMN had mutations in ABCA1 and MAP3K15. Combining the biological functions of these two genes, we speculated that the infiltration of dGCMN into the surrounding tissue may be due to the ablation of subcutaneous fat and the abnormal activation of MAPK pathway, which required further investigation.

## Conclusion

Here, we reported two cases of pediatric patients with different degrees of upper extremity GCMN development. We compared the genes of GCMN (both deep penetrating and non-penetrating tissues), small melanocytic nevus, normal skin and peripheral venous blood by WES analysis. Both patients showed mutations in NRAS, MUC16 gene in GCMN. Furthermore, by histological analysis, we suggest that the deep infiltrative character of GCMN may imply a high risk of malignant transformation, and at the genetic level, mutations in ABCA1 and MAP3K15 were found in dPGCMN. These novel mutated genes associated with GCMN may have potential value in predicting the risk of malignant transformation.

## Data Availability

Information supporting the data for the results reported in the article can be found in the submitted article.

## Abbreviations

GCMN: Giant congenital melanocytic nevus
MAPK: Mitogen-activated protein kinase
WES: Whole exome sequencing
ABCA1: ATP-binding cassette transporter A1
MAP3K15: Mitogen-activated protein kinase kinase kinase 15
SMN: Small melanocytic nevus
NS: Normal skin
PB: Peripheral venous blood
dPGCMN: Deep penetrating giant congenital melanocytic nevus
nPGCMN: Non-penetrating giant congenital melanocytic nevus

## References

[1] Viana AC, Gontijo B, Bittencourt FV (2013). Giant congenital melanocytic nevus. An Bras Dermatol.

[2] Tannous ZS, Mihm MC, Sober AJ, Duncan LM (2005). Congenital melanocytic nevi: clinical and histopathologic features, risk of melanoma, and clinical management. J Am Acad Dermatol.

[3] Kugar M, Akhavan A, Ndem I, Ollila D, Googe P, Blatt J, Wood J (2021). Malignant melanoma arising from a Giant congenital melanocytic nevus in a 3-year old: review of diagnosis and management. J Craniofac Surg.

[4] Watt AJ, Kotsis SV, Chung KC (2004). Risk of melanoma arising in large congenital melanocytic nevi: a systematic review. Plast Reconstr Surg.

[5] Krengel S, Hauschild A, Schäfer T (2006). Melanoma risk in congenital melanocytic naevi: a systematic review. Br J Dermatol.

[6] Aimaier R, Chung M, Zhu H, Yu Q (2022). Spatiotemporal expression of NRAS and occurrence of giant congenital melanocytic nevi. Exp Dermatol..

[7] Stark MS (2019). Large-Giant congenital melanocytic nevi: moving beyond NRAS mutations. J Invest Dermatol.

[8] Kumar R, Angelini S, Snellman E, Hemminki K (2004). BRAF mutations are common somatic events in melanocytic nevi. J Invest Dermatol.

[9] Wu D, Wang M, Wang X, Yin N, Song T, Li H, Yu J, Wang DM, Zhao Z (2011). Lack of BRAF(V600E) mutations in giant congenital melanocytic nevi in a Chinese population. Am J Dermatopathol.

[10] Krengel S, Scope A, Dusza SW, Vonthein R, Marghoob AA (2013). New recommendations for the categorization of cutaneous features of congenital melanocytic nevi. J Am Acad Dermatol.

[11] Cosgarea I, Griewank KG, Ungureanu L, Tamayo A, Siepmann T (2020). Deep penetrating nevus and borderline-deep penetrating nevus: a literature review. Front Oncol.

[12] Ruiz-Maldonado R, Tamayo L, Laterza AM, Durán C (1992). Giant pigmented nevi: clinical, histopathologic, and therapeutic considerations. J Pediatr.

[13] Wu M, Yu Q, Gao B, Sheng L, Li Q, Xie F (2020). A large-scale collection of giant congenital melanocytic nevi: clinical and histopathological characteristics. Exp Ther Med.

[14] Strazzula L, Senna MM, Yasuda M, Belazarian L (2014). The deep penetrating nevus. J Am Acad Dermatol.

[15] Garrido MC, Nájera L, Navarro A, Huerta V, Garrido E, Rodriguez-Peralto JL, Requena L (2020). Combination of congenital and deep penetrating nevus by acquisition of β-catenin activation. Am J Dermatopathol.

[16] Stark MS, Tell-Martí G, Martins da Silva V, Martinez-Barrios E, Calbet-Llopart N, Vicente A, Sturm RA, Soyer HP, Puig S, Malvehy J (2021). The distinctive genomic landscape of Giant congenital melanocytic nevi. J Invest Dermatol.

[17] Reinartz S, Failer S, Schuell T, Wagner U (2012). CA125 (MUC16) gene silencing suppresses growth properties of ovarian and breast cancer cells. Eur J Cancer.

[18] Wang X, Yu X, Krauthammer M, Hugo W, Duan C, Kanetsky PA, Teer JK, Thompson ZJ, Kalos D, Tsai KY (2020). The association of MUC16 mutation with tumor mutation burden and its prognostic implications in cutaneous melanoma. Cancer Epidemiol Biomark Prev.

[19] de Haan W, Bhattacharjee A, Ruddle P, Kang MH, Hayden MR (2014). ABCA1 in adipocytes regulates adipose tissue lipid content, glucose tolerance, and insulin sensitivity. J Lipid Res.

[20] Chung S, Sawyer JK, Gebre AK, Maeda N, Parks JS (2011). Adipose tissue ATP binding cassette transporter A1 contributes to high-density lipoprotein biogenesis in vivo. Circulation.

[21] Cuffe H, Liu M, Key CC, Boudyguina E, Sawyer JK, Weckerle A, Bashore A, Fried SK, Chung S, Parks JS (2018). Targeted deletion of adipocyte Abca1 (ATP-binding cassette transporter A1) impairs diet-induced obesity. Arterioscler Thromb Vasc Biol.

[22] Naguro I, Umeda T, Kobayashi Y, Maruyama J, Hattori K, Shimizu Y, Kataoka K, Kim-Mitsuyama S, Uchida S, Vandewalle A (2012). ASK3 responds to osmotic stress and regulates blood pressure by suppressing WNK1-SPAK/OSR1 signaling in the kidney. Nat Commun.

[23] Chen Z, Kong H, Cai Z, Chen K, Wu B, Li H, Wang P, Wu Y, Shen H (2021). Identification of MAP3K15 as a potential prognostic biomarker and correlation with immune infiltrates in osteosarcoma. Ann Transl Med.

